# Comparative Analysis of Erosion and Erosion-Abrasion Resistance of Bioactive Glass Ionomer-Based Restorative Materials: A Surface Characterization Study

**DOI:** 10.3390/biomimetics11030178

**Published:** 2026-03-03

**Authors:** Alaa Turkistani, Hanin E. Yeslam

**Affiliations:** 1 Department of Restorative Dentistry, Faculty of Dentistry, King Abdulaziz University, Jeddah 21589, Saudi Arabia; 2 Advanced Technology Dental Research Laboratory, King Abdulaziz University, Jeddah 21589, Saudi Arabia

**Keywords:** restorative materials, bioactive, glass ionomer cements, zirconium, wear, erosive challenges, roughness, SEM

## Abstract

Recently developed bioactive and reinforced glass ionomer cement (GIC) formulations may offer improved resistance to acid and mechanical wear compared to conventional formulations. Yet, comparative evidence under simulated oral conditions remains limited. This study evaluated the effect of erosive and erosive–abrasive challenges on the surface properties of five GIC-based restorative materials: Riva Self Cure (RS), Zirconomer Improved (ZI), Fuji II LC (FII), Equia Forte HT Fil + Equia Forte Coat (EQ), and ACTIVA BioACTIVE Restorative (AC). Standardized specimens from each material were immersed in artificial saliva, citric acid, or citric acid combined with simulated brushing. Surface roughness (Ra and Rq, µm) was measured, followed by qualitative surface characterization using scanning electron microscopy (SEM). Both material type and treatment condition significantly affected Ra and Rq values, with a significant interaction (*p* < 0.001). Erosive and erosive–abrasive challenges significantly increased surface roughness for all materials (*p* < 0.001). AC consistently exhibited the lowest values across all conditions, while ZI and RS showed the highest roughness, particularly under erosive–abrasive challenge. FII and EQ demonstrated intermediate performance. SEM observations corroborated profilometric findings, revealing material-dependent degradation patterns. All materials showed increased roughness following erosive and erosive–abrasive exposure. However, AC showed a comparatively more favorable profile than the other materials.

## 1. Introduction

Biomimetics refers to the application of nature-inspired concepts to reproduce the biomechanical and biological performance of natural tissues [1]. In restorative dentistry, this approach emphasizes the preservation of tooth structure and the replacement of lost tissues using materials and techniques designed to replicate the structure and function of the natural tooth [2,3]. Achieving this goal requires a thorough understanding of the microstructural, mechanical, and surface properties of enamel and dentin, as well as the selection of materials capable of forming a durable, seamless interface with the tooth [4,5,6]. Ideally, biomimetic material should also mimic the tooth’s natural self-repair potential [7]. This includes demonstrating bioactivity by eliciting a specific biological response and by releasing ions that promote remineralization and help prevent secondary caries, while maintaining sufficient mechanical strength to withstand intraoral conditions [2,3].

Glass ionomer cements (GICs) represent one of the earliest restorative materials designed to exhibit biomimetic characteristics [1]. GICs are formed through an acid–base reaction between fluoroaluminosilicate glass powders and aqueous polyalkenoic acid gel [8]. GICs are biomimetic because they match the tooth structure’s thermal expansion, form durable bonds to enamel and dentin, and release fluoride over time [1,9]. Their biocompatibility, chemical adhesion to tooth structure, and the ability to form a durable ion-exchange layer have supported their widespread clinical use, particularly in high-caries-risk patients [10,11]. Despite these advantages, conventional GICs exhibit notable limitations, including susceptibility to wear, low mechanical strength, prolonged setting times, and relatively rough surface morphology [10,12,13]. As a result, their clinical use has traditionally been limited in stress-bearing areas and chemically aggressive environments [10,13,14].

To address these limitations, modified and reinforced GIC-based formulations were developed by incorporating resin or silver alloy components, adjusting powder–liquid ratios, and optimizing polyacid chemistry to enhance matrix strength and mechanical performance [15]. Subsequent advancements focused on the incorporation of bioactive ionic resins, the application of protective surface coatings, and reinforcement with ceramic or zirconia fillers [13,16]. Previous studies have demonstrated that these modifications significantly enhanced mechanical strength, surface hardness, and wear behavior, resulting in more durable surface integrity and mechanical performance over time [17,18,19,20,21,22].

Among these developments, glass hybrid restorative systems such as EQUIA Forte and its optimized variant, EQUIA Forte HT, were introduced as advanced modifications of conventional GICs [23]. These glass hybrid systems employ a refined distribution of highly reactive glass particles within an enhanced polyacrylic acid matrix and are further supported by a nano-filled, light-curing surface coating to improve early moisture resistance and surface integrity [22]. According to the manufacturer, this modification enhances material reactivity and significantly improves mechanical properties, making the material suitable for long-term restorations in stress-bearing posterior regions. Previous studies have demonstrated improved compressive strength, wear resistance, and clinical longevity [15,22]. However, evidence regarding the material’s durability in low pH environments remains limited [24,25].

Activa Bioactive Restorative (AC), a resin–GIC hybrid material designed to bridge the gap between conventional GICs and resin-based composites [26]. The manufacturer describes the material as a bioactive, self-adhesive restorative material that promotes hydroxyapatite formation and interfacial remineralization via the release and recharge of calcium, phosphate, and fluoride ions, thereby supporting remineralization while maintaining enhanced physical performance [27]. AC contains a patented bioactive ionic resin matrix, a shock-absorbing resin component, and reactive ionomer glass fillers, designed to facilitate sustained release and recharge of calcium, phosphate, and fluoride ions while simultaneously enhancing toughness, resilience, and overall mechanical integrity [28]. From a compositional and mechanistic standpoint, AC has been described by several investigators as a reinforced or enhanced resin-modified GIC, as it incorporates a modified polyacid with limited water content and undergoes acid–base reactions similar to those observed in traditional GIC systems [16,29].

Zirconia-reinforced GICs, commonly referred to as “white amalgams,” were developed to enhance compressive and flexural strength while maintaining fluoride release [30,31]. The integration of zirconia particles into the glass matrix aims to reduce occlusal wear and improve fracture resistance [21,32]. While these materials demonstrate enhanced strength compared to conventional GICs, their surface behavior, especially under erosive and abrasive oral conditions, remains a subject of investigation [33,34].

Dental erosion, defined as the chemical dissolution of hard tissues and restorative materials by non-bacterial acids, has become increasingly prevalent due to modern dietary habits and lifestyle factors [35,36]. When erosion is combined with mechanical abrasion, the rate of surface degradation can be significantly accelerated [37]. Increased surface roughness is a clinical concern, as it predisposes restorations to plaque accumulation, staining, wear, and compromised esthetics, ultimately affecting restoration longevity and periodontal health [38,39,40]. Long-term clinical performance is therefore closely linked to the material’s ability to maintain a smooth, stable surface when exposed to fluctuating pH, dietary acids, and toothbrushing abrasion.

Accordingly, five GIC-based materials were selected to enable comparison between established glass ionomer systems and recently introduced formulations designed to improve resistance to surface degradation. Comparing these materials within a single standardized protocol offers clinically relevant insights into how differences in matrix chemistry, filler technology, and surface protection may influence resistance to erosive and erosive–abrasive challenges, an aspect that has been insufficiently addressed in the existing literature. Therefore, the present study aimed to comparatively evaluate their performance under simulated oral conditions by quantitatively measuring changes in surface roughness (Ra and Rq) and qualitatively analyzing surface morphology using scanning electron microscopy (SEM) following erosive and erosive–abrasive exposure. The null hypothesis was that neither material type, treatment condition, nor their interaction would result in statistically significant differences in surface roughness among the tested materials.

## 2. Materials and Methods

Five GIC–based restorative materials were selected for this study (Table 1): Riva Self Cure (RS; SDI Ltd., Bayswater, VIC, Australia), Zirconomer Improved (ZI; Shofu Inc., Kyoto, Japan), Fuji II LC (FII; GC Corp., Tokyo, Japan), Equia Forte HT Fil / Equia Forte Coat (EQ; GC Corp., Tokyo, Japan), and ACTIVA BioACTIVE Restorative (AC; Pulpdent Corp., Watertown, MA, USA). All materials were used strictly according to the manufacturers’ instructions.

### 2.1. Specimen Preparation

A total of 150 disc-shaped specimens (8 mm in diameter and 2 mm in thickness) were fabricated (*N* = 30 per material). Each material was packed into custom-made polytetrafluoroethylene molds, covered with Mylar strips, and pressed between glass slides to extrude excess material and ensure a smooth surface. For FII, AC, and Equia Forte Coat, polymerization was performed using an LED light-curing unit (AZDENT, Zhengzhou, China) with an irradiance of 1000 mW/cm^2^ on each surface (Table 1). After setting, specimens were removed from the molds and stored at 37 °C and 100% relative humidity for 24 h to allow controlled early maturation of the GIC.

Specimens were then polished using polishing discs (Sof-Lex; 3M Oral Care, Alexandria, MN, USA). For each polishing step, the flat surface of the disc was applied parallel to the specimen surface, with ten uniform strokes per application, each lasting approximately 2 s. Following completion of the polishing sequence, specimens were cleaned in an ultrasonic water bath to remove residual debris. To minimize operator-related variability, all specimen fabrication procedures were performed by the same operator.

The specimens were randomly allocated to three experimental groups (*n* = 10): the Control group, the Erosion group, and the Erosion/Abrasion group. Control specimens were stored in artificial saliva (pH 6.5) at 37 °C for the entire experimental period. The other groups underwent standardized erosive and erosive–abrasive cycling as described below [12,37].

For the erosion challenge, specimens were subjected to repeated immersion cycles in a 0.2 M citric acid solution (pH 2.3) for 5 min, followed by rinsing with distilled water and immersion in artificial saliva for 30 min. Each cycle concluded with a second distilled water rinse. This protocol was repeated six times daily over 5 days.

Specimens in the erosion–abrasion groups underwent the same erosive protocol, with simulated toothbrushing added after acid exposure. Brushing was performed using a toothbrushing simulator (SD Mechatronik, Feldkirchen-Westerham, Germany), applying 100 brushing strokes under a 1 N load with a medium-bristle toothbrush and a toothpaste–water slurry (Colgate Total Active Protection, Colgate-Palmolive, New York, NY, USA) prepared in a 1:3 ratio. After brushing, the specimens were rinsed with distilled water and immersed in artificial saliva for 30 min.

**Figure 1:**
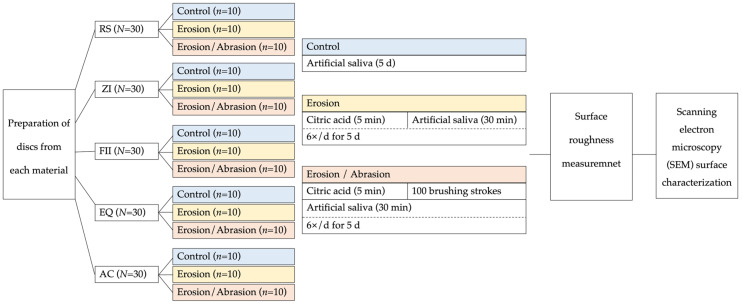
Schematic representation of the experimental design.

At the end of each day, specimens were stored at 37 °C in artificial saliva. Following completion of the experimental period, specimens were cleaned in an ultrasonic water bath, and profilometric analysis was conducted. A schematic summary of the experimental grouping and treatment conditions is shown in Figure 1.

### 2.2. Surface Roughness Measurement

Surface roughness was measured using a contact profilometer (Surftest SJ-210; Mitutoyo, Kawasaki, Japan) in accordance with ISO 12179:2021 standard [41]. The device was calibrated according to the manufacturer’s instructions. Three consecutive profilometric measurements were performed on each specimen surface using a cutoff length of 0.8 mm and an evaluation length of 5.0 mm. Average roughness (Ra) and root mean square roughness (Rq) (µm) were determined, and the mean value of three readings for each parameter was used for subsequent statistical analysis.

### 2.3. Scanning Electron Microscopy (SEM) Surface Characterization

Representative specimens were examined using a scanning electron microscope (AURA100, Seron Technologies, Uiwang-si, Republic of Korea) to observe surface morphology. The samples were sputter-coated with gold and examined at magnifications ranging from 100× to 2000×.

### 2.4. Statistical Analysis

The study used a 5 × 3 factorial design, involving five GIC-based restorative materials and three treatment conditions, yielding 15 experimental groups. A total sample size of 150 specimens (*n* = 10 specimens per group) was selected in the current study after a priori power calculation using G*Power software (version 3.1.9.6 for Mac OS, Heinrich-Heine-Universität Düsseldorf, Düsseldorf, Germany), assuming large effect sizes (Cohen’s *f* = 0.40), providing 96% power to detect large effects and adequate power (∼80%) to detect medium-to-large interaction effects at α = 0.05. This sample size allowed the detection of minimal clinically important differences of 0.2 μm in surface roughness [42].

Surface roughness parameters, RaRq, were used as dependent variables in the statistical analyses performed with R statistical software (Version 4.5.1, R Core Team, 2025, Vienna, Austria). All tests were two-tailed, and a significance level of α = 0.05 was applied. The Shapiro–Wilk test was used to confirm normality of the data across all experimental groups (all *p* > 0.05). Levene’s test was used to assess the homogeneity of variances assumption, and it revealed significant heteroscedasticity (*p* < 0.001). Therefore, Welch’s heteroscedasticity-corrected one-way analysis of variance (ANOVA) was used to detect the overall differences in Ra and Rq between materials pooled across treatments, and between treatment conditions pooled across materials. Material × treatment interactions were tested using heteroscedasticity-consistent (HC3) ANOVA. To test the effect of the treatment condition within each restorative treatment and the effect of material within each treatment condition, separate Welch’s ANOVA tests were conducted for each treatment condition and for each material. Games-Howell post hoc tests were conducted for pairwise comparisons with adjusted *p*-values using Welch-Satterthwaite degrees of freedom adjustment. All statistical tests were performed with a significance threshold of *p* < 0.05.

## 3. Results

### 3.1. Results for the Average Roughness (Ra)

#### 3.1.1. Effect of Material Within Each Treatment Condition

Within the control groups, Welch’s ANOVA revealed a significant effect of material on Ra, with effect *F* (4, 20.36) = 101.56 (*p* < 0.001). The lowest Ra values were detected in the AC and FII material groups. EQ showed statistically significantly greater Ra than FII (mean difference ≈ 0.147 µm, *p* < 0.001). ZI showed statistically significantly greater Ra than FII (mean difference ≈ 1.516 µm, *p* < 0.001). AC was significantly different than EQ (mean difference ≈ 0.160 µm, *p* < 0.001).

Within the erosion groups, a statistically significant effect of material on Ra was observed, with effect *F* (4, 21.37) = 314.58 (*p* < 0.001). AC) exhibited significantly lower Ra than FII (mean difference ≈ 0.587 µm, *p* < 0.001) and ZI (mean difference ≈ 2.498 µm, *p* < 0.001). EQ also showed significantly lower Ra than FII (mean difference ≈ 0.399 µm, *p* < 0.001).

Within the erosion/abrasion groups, Welch’s ANOVA revealed highly significant differences among materials, with effect *F* (4, 19.24) = 929.25 (*p* < 0.001). AC showed significantly lower Ra than FII (mean difference ≈ 0.833 µm, *p* < 0.001) and EQ (mean difference ≈ 1.520 µm, *p* < 0.001). EQ exhibited significantly lower Ra than FII (mean difference ≈ 0.687 µm, *p* < 0.001). The highest Ra values under erosion/abrasion were recorded for ZI (6.36 ± 0.60 µm) and RS (5.26 ± 0.61 µm).

#### 3.1.2. Overall Comparison Between Materials (All Treatments Combined)

Welch’s heteroscedastic ANOVA showed a statistically significant effect of material on Ra, *F* (4, 60.81) = 42.204, *p* < 0.001, ω^2^ = 0.715. When all treatment conditions were pooled, the materials ranked from lowest to highest mean Ra as follows: AC (0.318 ± 0.130 µm), FII (0.796 ± 0.479 µm), EQ (0.941 ± 0.758 µm), RS (3.150 ± 2.050 µm), ZI (3.630 ± 2.070 µm).

**Figure 2:**
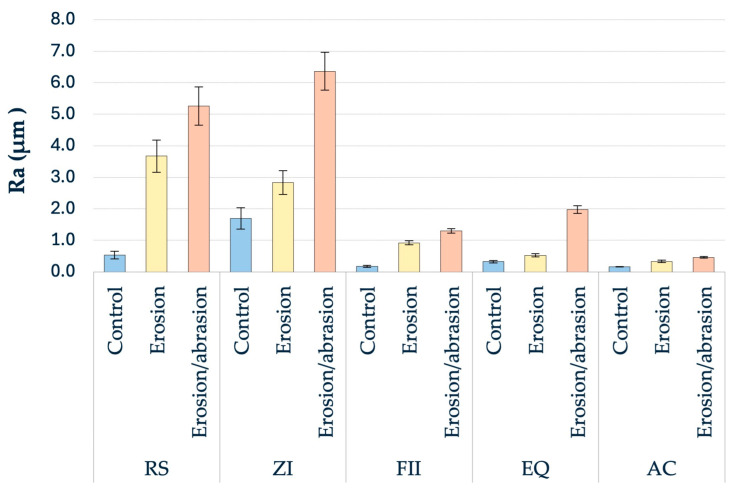
Bar graph illustrating the mean average surface roughness (Ra) values of the tested restorative materials following saliva storage, erosive challenge, and combined erosive–abrasive challenge. Error bars represent standard deviations.

Games–Howell post hoc tests indicated that AC exhibited significantly lower Ra than EQ, FII, RS, and ZI (all *p* < 0.01). EQ and FII did not differ significantly from each other (*p* > 0.05), but both showed significantly lower Ra than RS and ZI (all *p* < 0.001). No significant difference was detected between RS and ZI (*p* > 0.05). The descriptive statistics for Ra across all groups, including means and standard deviations, are detailed in Table 2 and graphically illustrated in Figure 2.

#### 3.1.3. Treatment Effects Within Each Material

For all materials, Welch’s ANOVA indicated a significant effect of treatment conditions on Ra (*p* < 0.001, ω^2^ ≥ 0.956). The ranking of treatment effects showed that erosive and erosive/abrasive conditions significantly increased surface roughness across all materials.

Within the AC groups, Ra increased significantly from control to erosion (and to erosion/abrasion. All pairwise comparisons were significant (*F* (2, 15.75) = 550.35, *p* < 0.001). Within the ZI groups, Ra increased significantly from control to erosion and was highest under erosion/abrasion (*F* (2, 17.28) = 220.99, *p* < 0.001, ω^2^ = 0.956). Within the EQ groups, the statistically significantly lowest Ra was observed in the control group, followed by erosion and erosion/abrasion (*F* (2, 16.20) = 900.99, *p* < 0.001, ω^2^ = 0.989). Within the FII material groups, Ra increased from control to erosion and erosion/abrasion (*F* (2, 15.35) = 1332.30, *p* < 0.001, ω^2^ = 0.993). Additionally, RS groups showed the lowest Ra in the control condition, whereas erosion and erosion/abrasion significantly increased Ra (*F* (2, 13.08) = 424.68, *p* < 0.001, ω^2^ = 0.981). All pairwise comparisons within treatment conditions were significantly different (*p* < 0.01).

#### 3.1.4. Interaction Between Material and Treatment for Ra

Type III two-way ANOVA showed significant main effects of material (sum of squares (SS) = 273.07, df = 4, mean square (MS) = 68.27, *F* = 787.23, *p* < 0.001, η^2^ₚ = 0.96) and treatment condition (SS = 157.32, df = 2, MS = 78.66, *F* = 907.07, *p* < 0.001, η^2^ₚ = 0.93). Additionally, there was a significant material × treatment interaction (SS = 101.06, df = 8, MS = 12.63, *F* = 145.67, *p* < 0.001, η^2^ₚ = 0.90).

### 3.2. Results of Root Mean Square Roughness (Rq)

#### 3.2.1. Material Comparisons Within Each Treatment Condition

Within the control groups, Welch’s ANOVA revealed significant between-material differences (*F* (4, 21.08) = 77.946, *p* < 0.001). Games–Howell comparisons showed significant differences between AC and EQ (diff ≈ 0.206 µm), AC and RS (≈0.691 µm), and AC and ZI (≈2.397 µm; all *p* < 0.05), confirming that AC surfaces are substantially smoother. This was also observed within the erosion groups (*F* (4, 20.32) = 148.804, *p* < 0.001), where AC presented significantly lower Rq than FII (mean difference ≈ 0.727 µm), RS (≈3.645 µm), and ZI (≈3.226 µm; all *p* < 0.05). For the erosion/abrasion condition, Welch’s ANOVA again indicated highly significant differences among materials (*F* (4, 18.59) = 869.463, *p* < 0.001). AC displayed the lowest Rq values, which were significantly lower than EQ (diff ≈ 2.111 µm), FII (≈1.332 µm), and RS (≈6.399 µm; all *p* < 0.05).

#### 3.2.2. Overall Comparison Between Materials and Between Treatment Conditions

Rq was significantly affected by the main effect material (Welch’s ANOVA, *F* (4, 62.34) = 42.578, *p* < 0.001, ω^2^ = 0.712). AC (0.469 ± 0.247 µm) ranked the lowest mean Rq across all treatments, followed by FII (1.16 ± 0.731 µm), EQ (1.262 ± 1.070 µm), then RS (4.047 ± 2.608 µm), and then ZI (4.879 ± 2.520 µm). When treatments were pooled across materials, mean Rq increased with the severity of the challenge: control (0.873 ± 0.956 µm), then erosion (2.11 ± 1.65 µm), and finally erosion/abrasion (4.11 ± 3.03 µm).

**Figure 3:**
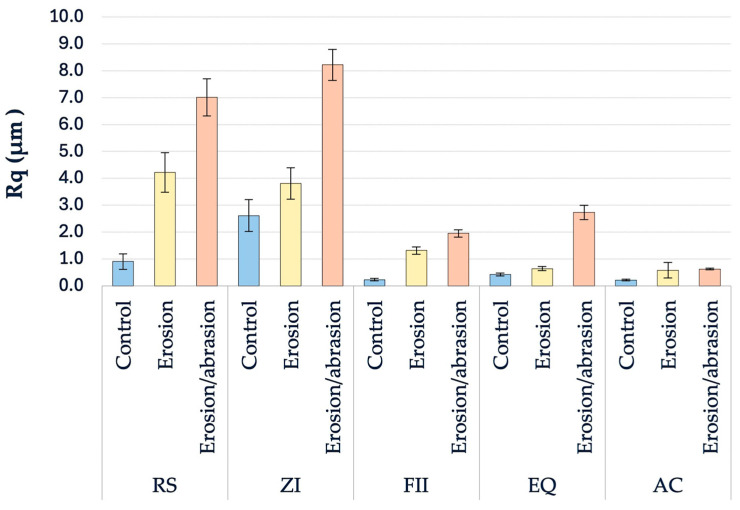
Mean root mean square surface roughness (Rq) of the evaluated restorative materials under different experimental conditions.

Games–Howell post hoc tests confirmed that AC exhibited significantly lower Rq than EQ, FII, RS, and ZI (*p* ≤ 0.003). While the mean Rq values of EQ and FII did not differ significantly (*p* > 0.05), both were significantly lower than those of RS and ZI (all *p* < 0.001). RS and ZI did not differ significantly in Rq (*p* > 0.05). The complete descriptive statistics of the Rq values in each group are detailed in Table 3 and illustrated in Figure 3.

#### 3.2.3. Treatment Effects Within Each Material

Within each material, Welch’s ANOVA indicated significant treatment effects on Rq (all *p* < 0.001, ω^2^ ≥ 0.959). Within the AC groups, Rq increased significantly (*F* (2, 15.96) = 412.811, *p* < 0.001, ω^2^ = 0.977) from control compared to erosion (*p* < 0.01) and to erosion/abrasion (*p* < 0.01). However, no significant difference was found between erosion and erosion/abrasion (*p* > 0.05). In ZI, Rq increased from the control to the erosion condition and was highest under erosion/abrasion. All pairwise differences were significant (*F* (2, 18) = 246.513, *p* < 0.001, ω^2^ = 0.959), with the largest increase between control and erosion/abrasion (mean difference ≈ 5.61 µm). Within the EQ groups, control also produced the lowest Rq, followed by erosion and erosion/abrasion. However, all pairwise comparisons were significant (*F* (2, 15.35) = 342.963, *p* < 0.001, ω^2^ = 0.974). This was also observed in the FII and RS groups (FII: *F* (2, 14.10) = 827.325, *p* < 0.001, ω^2^ = 0.990, RS: *F* (2, 15.03) = 361.007, *p* < 0.001, ω^2^ = 0.976), where Rq increased from control to erosion and erosion/abrasion with all pairwise comparisons were significant (*p* < 0.001).

#### 3.2.4. Interaction Between Material and Treatment for Rq

Type III two-way ANOVA indicated significant main effects of material (SS = 462.42, df = 4, MS = 115.61, *F* = 735.34, *p* < 0.001, η^2^ₚ = 0.96) and treatment condition (SS = 265.92, df = 2, MS = 132.96, *F* = 845.72, *p* < 0.001, η^2^ₚ = 0.93) on Rq, as well as a significant material × treatment interaction (SS = 144.74, df = 8, MS = 18.09, *F* = 115.08, *p* < 0.001, η^2^ₚ = 0.87).

### 3.3. Surface Characterization by SEM

**Figure 4:**
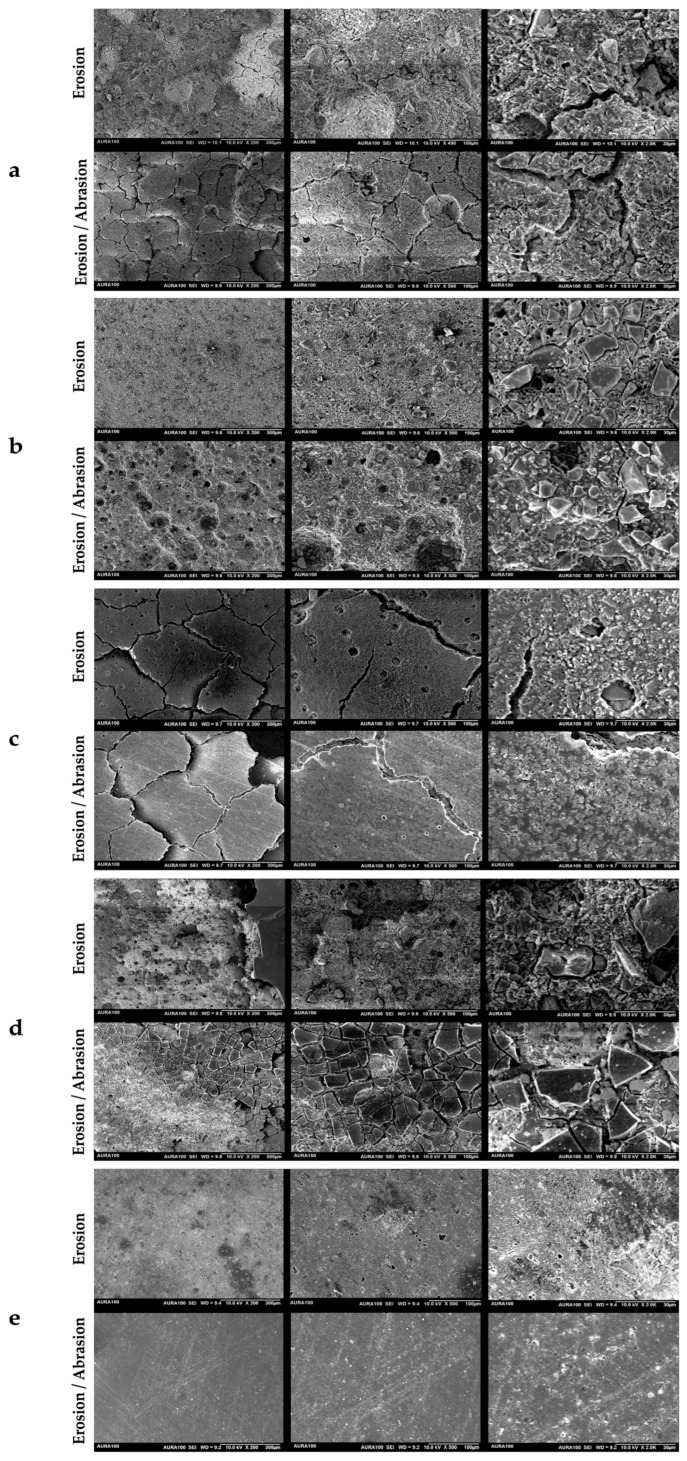
Representative scanning electron microscopy (SEM) images illustrating the surface morphology of the tested restorative materials at different magnifications (200×, 500×, and 2000×) following erosion and erosion–abrasion challenges: (**a**) Riva Self Cure (RS); (**b**) Zirconomer Improved (ZI); (**c**) Fuji II LC (FII); (**d**) EQUIA Forte HT/ Equia Forte Coat (EQ); and (**e**) ACTIVA BioACTIVE Restorative (AC).

Representative SEM micrographs revealed material-dependent surface morphological changes following erosive and erosive–abrasive challenges (Figure 4). Control specimens stored in artificial exhibited largely intact surfaces with minimal topographical alteration across all materials and were therefore excluded from presentation to allow clearer comparison of challenge-induced effects.

Following erosive challenge, SEM analysis of RS specimens revealed a heterogeneous surface characterized by matrix dissolution, scattered surface porosities, and crack formation. Higher magnification images revealed microvoids and protruding filler particles embedded within a degraded matrix. After the erosive–abrasive challenge, widespread cracking and surface-layer fragmentation were observed. At higher magnifications, extensive matrix loss, pronounced inter-particle gaps, and regions of particle dislodgement were evident, along with rough, eroded surface features.

Exposure to the erosive challenge resulted in superficial matrix dissolution in ZI specimens, with exposed, poorly supported filler particles, localized filler–matrix gaps, and microcracks. Under the erosive–abrasive challenge, the surface morphology was further modified by mechanical wear, showing extensive matrix loss with multiple large pits and voids across the surface. At higher magnifications, abrasion-related flattening and micro-texturing were evident, superimposed on an eroded substrate, while exposed filler-rich regions and multiple voids indicated continued matrix removal and filler detachment.

FII specimens subjected to acid erosion exhibited a surface characterized by crack formation and segmentation. At higher magnifications, the surface showed fine-scale heterogeneity, large voids, and filler exposure within the eroded matrix. After the erosive–abrasive challenge, fine, linear abrasion features aligned with the brushing direction were observed. The surface appeared more uniform, with fewer large voids and a relatively continuous matrix.

For EQ, specimens exposed to citric acid showed partial coating loss in several areas. SEM images revealed an exposed underlying glass hybrid material characterized by increased roughness and protruding fillers. Regions where the coating remained intact displayed comparatively smooth surfaces. In the erosive–abrasive group, coating loss was more extensive, and the exposed surfaces showed widespread fragmentation and cracking of the surface layer, with areas of complete coating removal and exposure of the underlying roughened glass ionomer substrate.

AC specimens exhibited a relatively homogeneous, compact surface morphology compared with those of other groups. The surface was characterized by fine-scale texture with scattered microvoids, exposed fillers, and isolated regions of localized degradation. After the erosive–abrasive challenge, the surface remained comparatively uniform with faint, linear abrasion features. At higher magnifications, the surface showed limited evidence of matrix disruption, with small, evenly distributed surface defects and dislodged filler particles. No evident surface cracks or voids were observed. After exposure to citric acid, the surface morphology remained largely intact, with only minor surface irregularities and limited filler exposure.

### 3.4. Summary of All Results

Across all analyses of Ra and Rq, both material type and treatment condition (control, erosion, erosion/abrasion) exhibited statistically significant effects as well as significant material × treatment interactions. AC consistently showed the lowest mean Ra and Rq values across all conditions, followed by FII and EQ. On the other hand, RS and ZI showed the highest roughness mean values, especially after exposure to erosive–abrasive challenges. Erosive and erosive-abrasive treatments significantly increased surface roughness across all materials compared with controls, and roughness increased systematically with increasing challenging conditions. Post hoc tests confirmed that AC had significantly lower roughness than all other materials; EQ and FII were similar to each other and smoother than RS and ZI; and RS and ZI did not differ significantly from each other overall.

SEM observations supported the profilometric data: RS and ZI showed extensive matrix dissolution, cracking, grooves, and filler loss after challenges; EQ exhibited progressive coating loss and exposure of a roughened substrate; FII showed cracks, voids, and abrasion marks but a relatively more continuous matrix after brushing; whereas AC maintained a comparatively homogeneous, compact surface with only minor textural changes and limited evidence of matrix disruption, even after erosive and erosive–abrasive exposures.

## 4. Discussion

The results of this study indicated that both material type and treatment condition, as well as their interaction, significantly influenced the surface roughness of the tested restorative materials, leading to rejection of the null hypothesis. Among all tested material–condition combinations, only AC and FII stored in saliva exhibited Ra values below the clinically relevant threshold of 0.2 µm. In contrast, exposure to erosive or erosive–abrasive challenges resulted in roughness values exceeding this threshold for all other materials. The highest roughness values were observed for ZI under erosion/abrasion, followed by RS under erosion/abrasion and under erosion. SEM revealed distinct, material-dependent surface morphologies that varied according to the challenge conditions. Overall, the SEM observations were consistent with the surface roughness measurements and provided qualitative insights into surface degradation following erosive and erosive–abrasive challenges.

In this study, all material specimens were finished and polished with a multi-step polishing system using impregnated discs (Sof-Lex; 3M Oral Care, Alexandria, MN, USA), known for producing superior results across many types of esthetic restorative materials, including GICs [43,44]. The same system was used for all specimens to ensure standardization and consistency across groups, reflecting a clinically relevant scenario in which clinicians may use a single polishing system for multiple materials without adjustments.

The experimental design aimed to simulate clinically relevant erosive and erosive–abrasive conditions under standardized laboratory settings. Citric acid was selected due to its frequent presence in acidic beverages, and the concentration and pH reflect their typical acidity. The cycling protocol and brushing regimen were adopted from established in vitro models to simulate repeated short-term dietary acid challenges followed by salivary recovery and the associated mechanical effects of toothbrushing, corresponding to approximately one year of twice-daily brushing [12,37].

RS showed extensive surface degradation, as evidenced by both profilometric measurements and SEM observations. This is consistent with its classification as a conventional chemically cured glass ionomer cement. Its matrix is formed exclusively through an acid–base reaction between polyacrylic acid and fluoroaluminosilicate glass, resulting in a hydrophilic, water-permeable structure [45]. The relatively large, irregularly shaped glass particles of GICs are more susceptible to partial dissolution during setting and subsequent acid exposure, which progressively reduces their support within the matrix [37]. This process is further intensified by dissolution of the peripheral siliceous hydrogel layer surrounding the glass particles, which compromises particle–matrix bonding and facilitates material loss. In addition, the inherent solubility of glass ionomer cements in aqueous environments promotes continuous water uptake and ion leaching, further weakening the matrix over time [46]. The presence of porosity and microcracks enhances fluid diffusion, accelerating these degradation mechanisms [15]. Even under saliva storage, ongoing post-setting maturation with ion exchange may contribute to increased surface roughness. Under a citric acid challenge, pronounced dissolution of the glass particles, leaching of ions, and surface softening are expected, leading to increased roughness [47]. When brushing is added, the softened matrix and poorly supported fillers are readily removed, resulting in pronounced surface degradation. These findings reinforce the well-documented limitations of conventional GICs in acidic and high-wear environments [13].

ZI also demonstrated unfavorable performance, despite its zirconia reinforcement. It exhibited the highest Ra and Rq values under most conditions, which is consistent with the pronounced surface degradation observed in the SEM micrographs. Similar to RS, ZI is fundamentally based on an acid–base reaction between polyacrylic acid and a fluoroaluminosilicate glass matrix, and therefore retains an intrinsically hydrophilic, water-permeable polysalt structure. The incorporation of zirconia particles results in a heterogeneous filler system composed of acid-reactive glass particles and chemically inert zirconia fillers [21]. As zirconia fillers do not participate in the acid–base setting reaction, dissolution of glass particles and the surrounding polysalt matrix under aqueous and acidic conditions leads to preferential exposure of zirconia particles. This mismatch may compromise filler–matrix cohesion, particularly if bonding between zirconia particles and matrix is suboptimal [20]. As matrix dissolution progresses, zirconia particles become increasingly poorly supported and susceptible to dislodgement, especially under abrasive brushing. In addition, inherent and air-entrapped porosity from hand mixing, together with microcracks, may further compromise structural integrity, as crack propagation along filler–matrix interfaces can interconnect pores and facilitate water and acid diffusion, thereby accelerating surface degradation [48]. Furthermore, materials containing larger filler particles are generally characterized by rougher surfaces, due to the particle size’s influence on GIC surface roughness [49,50,51]. Previous reports have indicated that ZI’s filler particles are larger than those of other GICs [33,50]. These findings indicate that zirconia reinforcement in conventional GICs may enhance bulk properties but does not necessarily improve surface stability and may instead exacerbate surface roughening under erosive and erosive–abrasive conditions [33,52,53].

FII, as a resin-modified GIC, demonstrated intermediate performance, reflecting its dual-setting mechanism and hybrid matrix structure. The presence of hydroxyethylmethacrylate (HEMA) enables light-activated polymerization, improves early strength, and reduces early moisture sensitivity. Nevertheless, its hydrophilic nature promotes water sorption during storage and acidic exposure [37]. In addition, FII still contains a substantial ionomer phase, which remains vulnerable to acidic attack. Exposure to citric acid is therefore expected to induce matrix softening and disruption of the filler–matrix interface, while subsequent abrasive brushing can preferentially remove these weakened regions, leading to filler debonding and the development of surface irregularities [54,55]. As a result, although FII performs better than self-cured RS and ZI, it does not reach the level of stability observed with resin-dominant AC.

EQ is a glass hybrid restorative material that combines a highly reactive fluoroaluminosilicate glass with ultrafine hybrid fillers and a light-cured protective resin coating. This formulation promotes dense filler packing and improved mechanical and surface properties compared with conventional GICs. Previous studies have demonstrated that EQUIA Forte exhibits significantly higher hardness and wear resistance than conventional high-viscosity GICs, indicating a more resistant matrix–filler structure [15,22]. The light-cured surface coating functions as an effective barrier, sealing surface porosities and restricting water sorption, thereby improving surface characteristics [19]. However, SEM observations indicated fragmentation or partial degradation of the resin coating under erosive and erosive-abrasive challenges, exposing the underlying ionomer matrix to chemical attack. Brushing may have accelerated coating loss and promoted filler exposure and plucking, leading to increased roughness [25]. In addition, the presence of abrupt transitions between coated and uncoated regions, together with cracks and fragmented coating edges, contributed to a highly heterogeneous surface topography. Loss of coating integrity reduces surface protection and exposes the underlying acid–base matrix, which remains inherently susceptible to erosion. Previous comparative studies reported no statistically significant differences in erosive wear between uncoated EQ and conventional GICs, suggesting that reductions in particle size and increased glass reactivity may have a limited influence on resistance to acid-mediated degradation [24].

AC consistently demonstrated the most favorable surface characteristics across all experimental conditions. This can be explained by its distinctive chemistry and dual-setting mechanism, which place it at the interface between resin composites and resin-modified glass ionomer cements. AC features a resin-dominant matrix composed of urethane methacrylate–based monomers combined with polyacrylic acid, enabling the formation of two interpenetrating networks through concurrent resin polymerization and a limited acid–base reaction [16]. In addition, the incorporation of silanized fluoroaluminosilicate glass and silanized nonreactive fillers promotes effective filler–matrix coupling, contributing to enhanced wear resistance and preservation of surface integrity [56]. The relatively fine, homogeneously dispersed fillers promote a more uniform stress distribution and reduced interparticle spacing, which may limit filler pull-out and contribute to improved wear resistance [55,57,58].

Nevertheless, AC still showed a significant increase in surface roughness following erosive and erosive–abrasive challenges relative to saliva storage. This can be attributed to the presence of a polyacid-containing phase and a limited acid–base reaction, which permit superficial ion release and partial softening of the ionic network under acidic exposure. Moreover, acidic environments have been shown to adversely affect resin-based materials by inducing chemical degradation of the organic matrix, resulting in surface softening and leaching of unreacted monomers [45]. Water sorption likely further contributed to the observed roughness increase, as the resin matrix of AC contains hydrophilic components, which facilitate water uptake and acid diffusion within the material [55,59]. This superficial chemical conditioning likely increased susceptibility to mechanical wear, resulting in higher roughness. Nevertheless, the observed degradation remained confined to the surface and did not involve the extensive matrix dissolution or filler destabilization as observed in the other material groups. These findings are consistent with the classification of AC as a reinforced resin-modified glass ionomer–like material, in which resin polymerization dominates surface behavior while residual ionomer chemistry explains its comparatively limited sensitivity to erosive and erosive–abrasive conditions.

One limitation of the present study is the use of a single polishing protocol for all materials. This standardized approach may have disadvantaged higher-viscosity and zirconia-reinforced GICs, which may not achieve the same level of surface smoothness as other materials when finished with the same polishing protocol [43]. Further research is recommended to examine how various polishing systems affect these materials under erosive and abrasive conditions, including an initial SEM assessment. In addition, advanced analytical techniques, such as atomic force microscopy and nanoindentation, may provide further insight into nanoscale surface changes and mechanical behavior following degradation. Finally, in situ and clinical studies are essential to validate these in vitro findings and to determine their long-term clinical implications in patients with high erosive risk.

## 5. Conclusions

Within the limitations of this in vitro study, the resin-modified glass ionomer hybrid AC demonstrated the most favorable surface stability following erosive and erosive–abrasive challenges. In contrast, conventional and zirconia-reinforced GICs were more susceptible to degradation. Further in situ and clinical studies are required to validate these findings.

## Figures and Tables

**Table 1:** The composition and manufacturers of glass ionomer–based restorative materials were evaluated.

Material (Code)	Manufacturer	Category	Main Components	Application
Riva Self Cure (RS)	SDI Ltd., Bayswater, VIC, Australia	Conventional glass ionomer cement	Powder: Fluoroaluminosilicate glass Liquid: Acrylic acid homopolymer, tartaric acid, water	Capsule activated and mixed (10 s)
Zirconomer Improved (ZI)	Shofu Inc., Kyoto, Japan	Zirconia- reinforced glass ionomer	Powder: Fluoroaluminosilicate glass, zirconium oxide Liquid: Polyacrylic acid solution, tartaric acid	Hand mixed powder and liquid; total mixing time ≤30 s
Fuji II LC (FII)	GC Corp., Tokyo, Japan	Resin modified glass ionomer cement	Powder: Fluoroaluminosilicate glass Liquid: 2-hydroxyethylmetacrylate (HEMA), polyacrylic acid, polybasic carboxylic acid, dimethacrylate, urethane dimethacrylate (UDMA), water, photoinitiator	Capsule activated and mixed (10 s); light-cured (20 s)
Equia Forte HT Fil / Equia Forte Coat (EQ)	GC Corp., Tokyo, Japan	Glass hybrid restorative system with surface coating	Powder: Fluoroaluminosilicate glass, ultrafine hybrid glass fillers, polyacrylic acid powder Liquid: Polyacrylic acid, polybasic carboxylic acid, water light-cured coating resin: Methyl methacrylate, photoinitiator, synergist, phosphoric acid ester monomer, butylated hydroxytoluene (BHT)	Capsule activated and mixed (10 s); coat applied and light-cured (20 s)
ACTIVA BioACTIVE Restorative (AC)	Pulpdent Corp., Watertown, MA, USA	Bioactive resin-modified glass ionomer hybrid	Diurethane and other methacrylates with modified polyacrylic acid, amorphous silica, photoinitiator, sodium fluoride	Dispensed; self-cured (20 s) and light cured (20 s)

**Table 2:** Descriptive statistics of the average roughness (Ra) results across all experimental conditions and tested materials.

Material	Treatment Condition	Mean	Std. Deviation	Minimum	Maximum	95% CI for Mean
RS	Control	0.53	0.12	0.38	0.73	0.44–0.61
	Erosion	3.67	0.51	3.08	4.77	3.30–4.03
	Erosion/abrasion	5.26	0.61	4.33	6.38	4.83–5.70
ZI	Control	1.69	0.34	1.23	2.21	1.45–1.93
	Erosion	2.83	0.38	2.37	3.53	2.56–3.10
	Erosion/abrasion	6.36	0.60	5.13	7.12	5.93–6.79
FII	Control	0.17	0.03	0.14	0.22	0.15–0.19
	Erosion	0.92	0.06	0.79	0.99	0.87–0.96
	Erosion/abrasion	1.30	0.07	1.19	1.39	1.24–1.35
EQ	Control	0.32	0.04	0.28	0.41	0.29–0.35
	Erosion	0.52	0.05	0.45	0.62	0.48–0.55
	Erosion/abrasion	1.98	0.12	1.81	2.21	1.90–2.070
AC	Control	0.16	0.01	0.14	0.19	0.15–0.17
	Erosion	0.33	0.04	0.28	0.39	0.30–0.36
	Erosion/abrasion	0.46	0.03	0.42	0.49	0.45–0.48

**Table 3:** Descriptive statistics of the root-mean-square roughness (Rq) results across all treatment conditions and tested materials.

Material	Treatment Condition	Mean	Std. Deviation	Minimum	Maximum	95% CI for Mean
RS	Control	0.90	0.29	0.56	1.32	0.70–1.11
	Erosion	4.22	0.74	3.65	6.26	3.69–4.76
	Erosion/abrasion	7.01	0.69	5.79	7.87	6.52–7.51
ZI	Control	2.61	0.59	1.64	3.63	2.19–3.03
	Erosion	3.81	0.58	2.94	4.91	3.39–4.22
	Erosion/abrasion	8.22	0.58	7.00	8.88	7.81–8.64
FII	Control	0.23	0.04	0.16	0.32	0.19–0.26
	Erosion	1.31	0.14	1.07	1.49	1.21–1.40
	Erosion/abrasion	1.95	0.14	1.68	2.21	1.85–2.05
EQ	Control	0.42	0.05	0.37	0.54	0.38–0.45
	Erosion	0.64	0.08	0.50	0.77	0.59–0.70
	Erosion/abrasion	2.73	0.27	2.31	3.2	2.53–2.92
AC	Control	0.21	0.03	0.19	0.29	0.19–0.23
	Erosion	0.58	0.29	0.38	1.21	0.37–0.79
	Erosion/abrasion	0.62	0.03	0.55	0.66	0.59–0.64

## Data Availability

The raw data supporting the conclusions of this article will be made available by the authors on request.

## Abbreviations

The following abbreviations are used in this manuscript:

AC	ACTIVA BioACTIVE Restorative
ANOVA	Analysis of variance
EQ	EQUIA Forte HT
FII	Fuji II LC
GIC	Glass ionomer cement
HEMA	Hydroxyethylmethacrylate
RS	Riva Self Cure
ZI	Zirconomer Improved
